# Transanal formation of anastomosis using C-REX device is feasible and effective in high anterior resection

**DOI:** 10.1007/s00384-023-04420-x

**Published:** 2023-05-13

**Authors:** Dadi Vilhjalmsson, Mattias Lepsenyi, Ingvar Syk, Anders Grönberg, Henrik Thorlacius

**Affiliations:** 1 Department of Clinical Sciences, Section of Surgery, Malmö, Lund University, Malmö, 205 02 Sweden; 2 Section of Development, CarpoNovum, Halmstad, 302 41 Sweden

**Keywords:** Anastomosis, Colorectal, Compression, Healing, CARP, C-REX

## Abstract

**Purpose:**

C-REX is a novel instrument for creating stapleless colorectal anastomosis by compression. The aim of this study was to evaluate the feasibility and effectiveness of C-REX in open and laparoscopic high anterior resections.

**Methods:**

A prospective clinical safety study on 21 patients reconstructed with C-REX colorectal anastomosis following high anterior resection of the sigmoid colon using two different devices for intraabdominal (*n* = 6) or transanal (*n* = 15) placement of the anastomotic rings. Any signs of complications were prospectively monitored by a predefined protocol. Anastomotic contact pressure (ACP) was measured via a catheter-based system, and time for evacuation of the anastomotic rings by the natural route was noted. Blood samples were collected daily, and flexible endoscopy was performed postoperatively to examine macroscopic appearance of the anastomoses.

**Results:**

One of six patients operated with the intraabdominal anastomosis technique with an ACP of 50 mBar had to be reoperated because of anastomotic leakage. None of the 15 patients operated with the transanal technique (5 open and 10 laparoscopic procedures) had anastomotic complications, and their ACP ranged between 145 and 300 mBar. C-REX rings were uneventfully expelled by the natural route in all patients after a median of 10 days. Flexible endoscopy showed well-healed anastomoses without stenosis in 17 patients and a moderate subclinical stricture in one patient.

**Conclusion:**

These results indicate that the novel transanal C-REX device is a feasible and effective method for colorectal anastomosis following high anterior resections, irrespective of open or laparoscopic approach. Moreover, C-REX allows measurement of intraoperative ACP and thereby a quantitative evaluation of the anastomotic integrity.

**Supplementary Information:**

The online version contains supplementary material available at 10.1007/s00384-023-04420-x.

## Introduction

Stapel devices were first introduced in surgery by Hümer Hültl in 1908 and Aladár Petz in 1924 [1]. More refined stapling instruments were developed in Russia in the fifties, and these instruments were later modified further by Mark Ravitch in the 1970s to facilitate colorectal anastomoses [2, 3]. Since then, several modifications have been undertaken, and circular stapling devices constitute today a cornestone in rectal cancer surgery. However, anastomosis-related complications are still a major problem in stapled colorectal anastomoses. Anastomotic leakage (AL) is the most feared one, occurring in approximately 10–20% [4–10] and has severe impact on both patient recovery and oncological outcome [11–15]. The cause of leakage is most likely multifactoral, depending on complex interactions between the surgical procedure and patient characteristics. Technical failures of the anastomosis due to surgical disruption of the blood supply or tension at the anastomotic site are well-known causes of leakage [16]. Another potential cause is related to foreign body reaction in the anastomosis as sutures and staplers cause local inflammation which can disturb healing through excessive breakdown of collagen [17–20].

As an alternative to stapler devices, anastomotic compression devices have been developed, which leave no foreign body material in the anastomosis [20–24]. Among them are the most well-known Murphy’s button and Valtrac BAR, of which the Valtrac BAR has been reported in numerous studies as feasible and safe, but is cumbersome to use and has been abandoned [21–27]. Recently, a novel compression anastomotic device called CARP (compression anastomotic ring-locking procedure) has been introduced [28]. CARP allows not only a stapel-free anastomosis, but has also four built-in catheters enabling measurement of the intraoperative anastomotic contact pressure (ACP) and postoperative monitoring of potential leakage [28, 29]. This makes CARP a unique device with capacity to quantify anastomotic integrity. A previous study showed that CARP was effective and safe in left-sided colo-colic anastomoses in patients undergoing resection of the sigmoid colon [29]. However, the CARP device is limited to open surgery and has now been modified in order to be used in minimally invasive procedures and for transanal rectal anastomoses. This new instrument is named C-REX (colorectal anastomosis; rejoin the intestine and validate the anastomosis; extract samples for analysis; X-ray through connected catheters) and includes one device adopted for intraabdominal anastomoses (LapAid with LapAid, LL) and one device adopted for transanal anastomoses (LapAid with RectoAid, LR).

Based on the considerations above, the aim of this study was to evaluate the safety and efficacy of C-REX (LL and LR) in open and laparoscopic surgery and transanal formation of colon-to-rectum anastomosis in high anterior resection.

## Material and methods

### Study design

A non-randomized prospective safety study on patients undergoes open or laparoscopic high anterior resection with a colorectal anastomosis below the sacral promontory 10–12 cm above the anal verge. The inclusion criteria were patients ≥ 18 years undergoing resection due to confirmed or suspected adenocarcinoma in the sigmoid colon, having a cognitive ability to take part in the study and understand oral and written study information. All patients signed a written consent of participation. Exclusion criteria were American Society of Anesthesiologists (ASA) score ≥ IV, albumin level < 25 g/L, stage IV colorectal cancer, emergency surgery, concurrent inflammatory bowel disease, or treatment with immunosuppressive medications (other than NSAIDs) less than 4 weeks prior to surgery. All procedures were conducted in accordance with the Helsinki declaration on ethical principles for medical research involving humans. The study was approved by the Regional Ethical Committee at Lund University, Sweden (2019–00,978).

### Patients

Twenty-one patients (16 males and 5 females) were included. Oral bowel preparation was performed the day before surgery with 2 L of sodiumpicosulphate solution (Picoprep, Ferring Läkemedel AB, Malmö, Sweden). Hospital routines according to a local ERAS concept were followed regarding antibiotic prophylactics (preoperative 1.5 g metronidazole and 800 mg sulfamethoxazole/160 mg trimethoprim orally), postoperative pain management (epidural anesthesia in open surgery and spinal anesthesia in laparoscopic surgery), and postoperative mobilization. However, the patients received only liquid diet the first 5 postoperative days. Patient demographics were collected, including ASA score, comorbidities, and body mass index (BMI). Ligation of arteria mesenterica inferior was performed below the left colic artery branch in all patients. The size of the instrument used was recorded, as was the type of pairs of the C-REX device used (LapAid with LapAid (LL) for intraabdominal use or LapAid with RectoAid (LR) for transanal use). Thus, two different device systems (LL and LR) were used for intraabdominal and transanal formation of colorectal anastomoses. ACP was measured three times after completion of the anastomosis, and the mean pressure value was used. Daily postoperative blood samples were collected for analysis of white blood cell (WBC) counts and C-reactive protein (CRP). The duration of surgery, time to return of bowel function, and time for expulsion of the anastomotic C-REX rings by the natural route were noted. Any adverse events, including AL, were recorded. The length of stay was predetermined to be 6 days according to study protocol. All patients were followed up 4 to 5 weeks after surgery for clinical evaluation and information on pathological findings. Postoperative adjuvant treatment was given according to local routines. In addition, macroscopic appearance of the anastomosis was examined by flexible endoscopy 4–25 weeks postoperatively.

### C-REX device

**Fig. 1:**
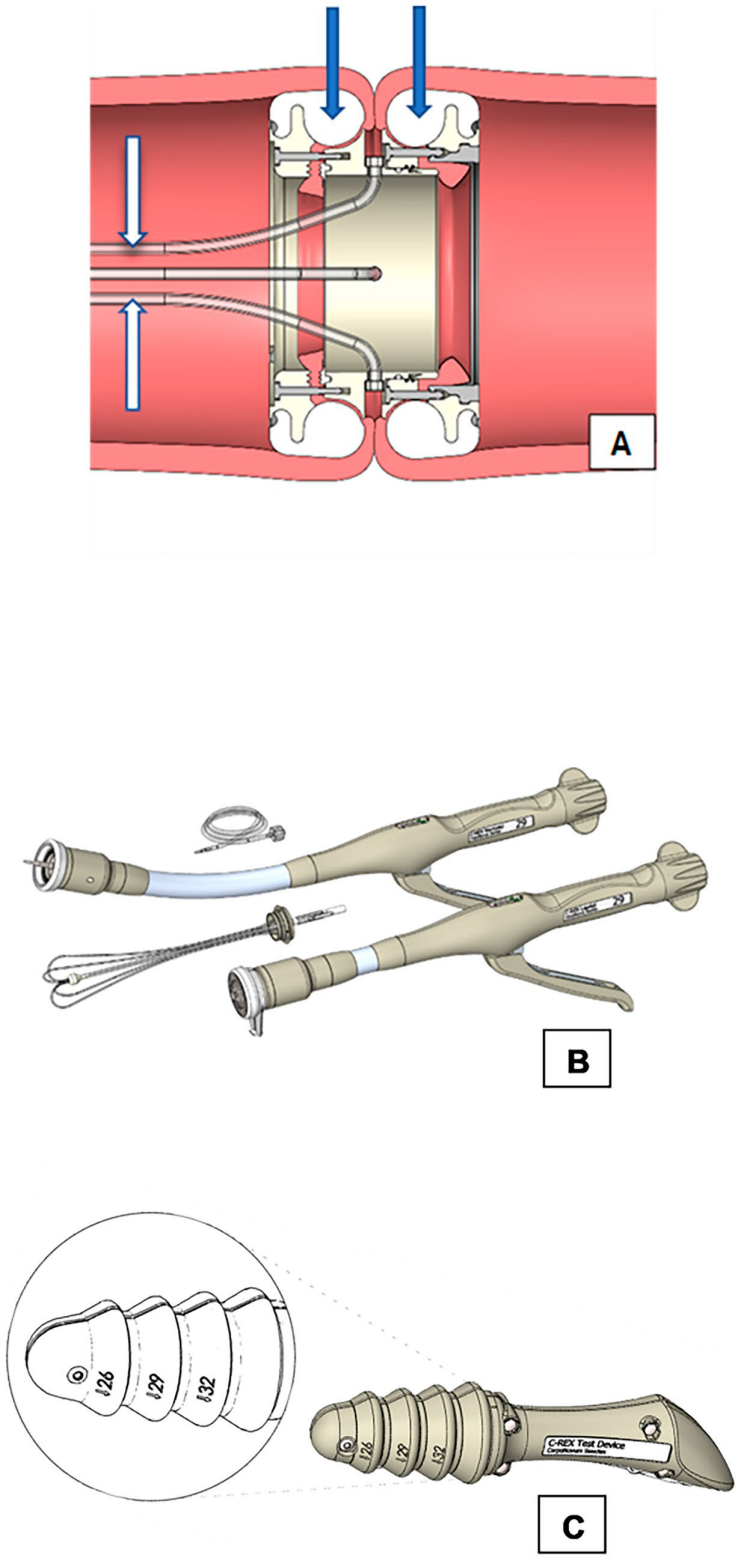
A cross section of a C-REX colorectal anastomosis after the RectoAid instrument has connected the proximal and distal C-REX rings together (**A**). The bowel wall is anchored between the silicon rings (blue arrows) and the anastomotic rings. The C-REX anastomosis contains four built-in catheters (white arrows) in connection to the anastomotic space, running from the anastomosis via the distal bowel segment and protruding through the anus. This catheter system enables measurement of the intraoperative anastomotic contact pressure and postoperative monitoring of the anastomotic integrity. The middle figure shows the LapAid-RectoAid pairs of the C-REX device and their associated catheters (**B**). The RectoAid instrument is similar in appearance as the circular staple instrument with its curved design. The lower figure shows the specific test device used for measuring the best suitable mean size of the C-REX rings (26, 29, and 32 mm) during the anastomotic construction (**C**)

**Fig. 2:**
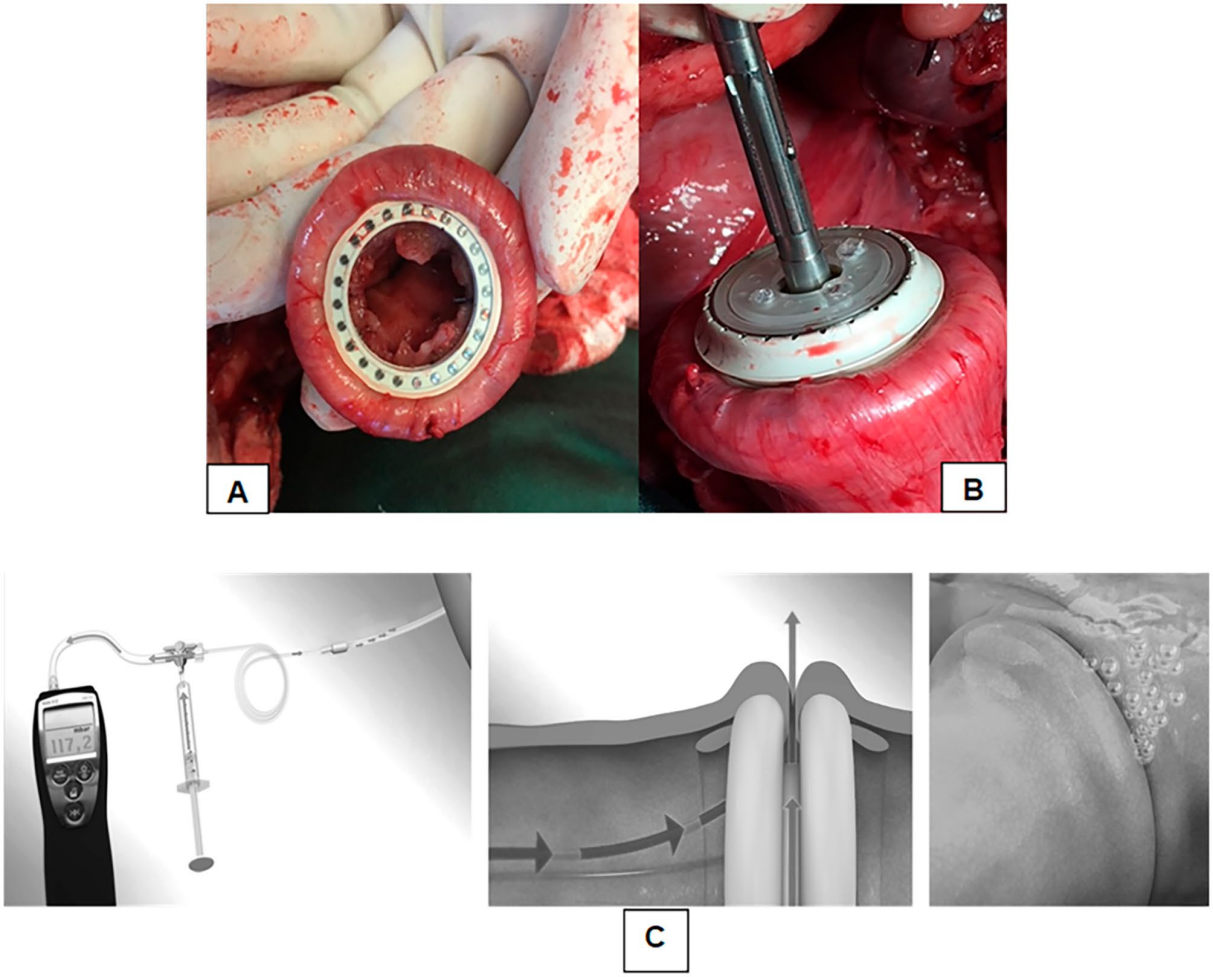
The C-REX ring in place in the proximal bowel end after its placement with the help of the LapAid instrument (**A**). The picture shows the proximal C-REX ring before and after the insertion of the anvil part (**B**). ACP is determined by infusing air via the catheter system and simultaneously measuring of the contact pressure (**C**). Appearance of air bubbles from the submerged anastomosis indicates ACP

The C-REX device is a refined CARP device [28, 29] enabling the use of minimally invasive surgery as well as transanal construction of a colorectal anastomosis. In brief, the C-REX is a snap-locking device, where the bowel ends are held together by a proximal and a distal ring. The C-REX anastomosis forms an oval contact surface area separated from the point of necrosis to increase the healing area in the anastomosis (Fig. 1A). As shown in the figure, the C-REX device contains four built-in catheters in connection to the anastomotic space, running from the anastomosis via the distal bowel segment and protruding through the anus (3 of 4 catheters visible in the figure). This catheter system enables measurement of the intraoperative ACP without X-ray as well as postoperative monitoring of anastomotic integrity with X-ray. When constructing a C-REX anastomosis, the two bowel ends are folded around the anastomotic rings, which are snapped into the C-REX device. There are two specific instruments for placing the anastomotic rings into the bowel lumen, the LapAid instrument to be used intraperitoneally, and the RectoAid instrument for transanal use (Fig. 1B), and also a specific test device for measuring the best suitable size of the C-REX rings (Fig. 1C). These instruments are used in pairs when constructing the anastomosis. For totally intraabdominal construction, LapAid instrument is used for placement of anastomotic rings in both bowel ends (LL-technique). In transanal anastomosis construction, LapAid is used for the proximal bowel end, whereas the RectoAid instrument is used transanally (LR-technique) (Supplementary Fig. 1). RectoAid is similar in appearance to circular stapler devices used in common practice today with an anvil to be placed in the proximal bowel end and connected to the transanally introduced instrument (Fig. 2A and B, and Supplementary Fig. 1). The staple lines in the transected bowel ends are removed before placement of the C-REX rings, and the staple line in the transected rectal stump should be carefully invaginated into the distal anastomotic C-REX ring if possible and thus removed at firing of the RectoAid instrument. After construction of the C-REX anastomosis, the ACP is determined by infusing air into the catheters while simultaneously measuring the pressure with a manometer until infused air starts to leak from the anastomosis, creating an abrupt drop in pressure defined as intraoperative ACP (Fig. 2C). Anastomotic integrity can be determined by radiology postoperatively by infusing water soluble contrast via the catheters [28, 29].

**Fig. 3:**
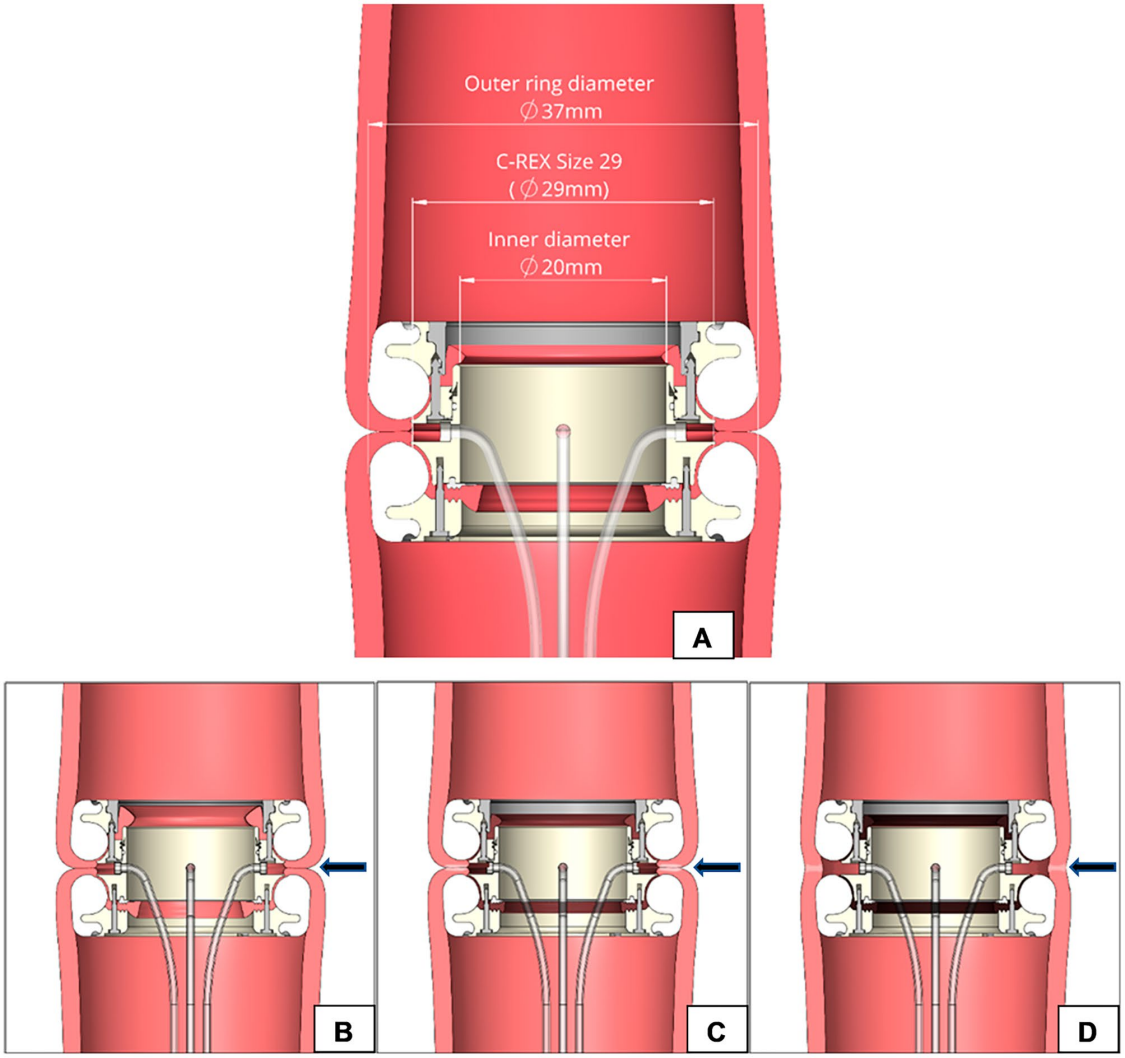
The CREX-device exists in three different mean sizes of 26, 29, and 32 mm. The 29-mm C-REX device creates an anastomosis with an inner diameter of 20 mm when the anastomotic rings are still in place, but has an outer diameter of 37 mm (**A**). When the anastomotic rings have detached, the C-REX anastomosis gains its final shape and acquires a lumen that corresponds closer to the outer diameter of the anastomotic rings. **B**–**D** The timeline of the healing process with progressing necrosis of the bowel wall being compressed between the anastomotic silicon rings (black arrows), until the rings detach from the newly constructed anastomosis and are expelled through the natural route. The C-REX anastomosis forms an oval contact surface area separated from the point of necrosis to increase the healing area in the anastomosis

The CREX-device is made in three different mean sizes (26, 29, and 32 mm). The 29-mm C-REX device, for example, creates an anastomosis with an inner diameter of 20 mm when the anastomotic rings are still in place, but has an outer diameter of 37 mm, hence the mean size diameter of 29 mm (Fig. 3A). When the anastomotic rings detach during the healing process by necrosis, the C-REX anastomosis theoretically gains its final shape and acquires a lumen that corresponds closer to the outer diameter of the anastomotic rings (Fig. 3B–D).

### Statistics

Non-parametric statistical methods and concepts were used, and range, medians, and quartiles (*Q*) are presented.

## Results

Twenty-one patients were included and anastomosed with the C-REX devices. The first 11 patients were operated with open approach and the following 10 patients laparoscopically. One laparoscopic procedure was converted to open surgery due to extensive adhesions. The first 6 patients were anastomosed intraabdominally with the LL-technique and the following 15 patients transanally with the LR-technique.

Patient age ranged between 46 and 85 years (median 72). BMI varied between 18 and 30 (median 26). Three patients had an ASA score of 1, fourteen a score of 2, and four a score of 3. The most common comorbidities included hypertension (9 patients), cardiovascular disease (7 patients), and diabetes mellitus (4 patients) (Table 1).

**Table 1:** Patient characteristics

**Patient**	**ASA score**	**BMI**	**Comorbidities**	**T-stage**
1	2	22	HTN, DM	T3c
2	1	18	Hypothyroidism	T3b
3	2	26	None	T3b
4	2	29	HTN, AF, TIA	T3c
5	3	28	AF, CVI	T3b
6	2	30	HTN	T1sm2
7	2	26	DM, TIA	T3a
8	2	29	None	T4a
9	3	25	HT, AF	Adenoma
10	2	27	HTN	T4a
11	2	28	DM	T2
12	2	25	Hypothyroidism	T3b
13	2	28	HTN	T3a
14	2	22	Temporal arteritis	T2
15	2	27	None	T2
16	1	24	None	T3a
17	3	24	HTN, AF, TIA	Adenoma
18	2	26	HTN, DM, single kidney	T4a
19	1	27	None	T1sm2
20	2	25	HTN	T3b
21	3	22	AF	T1sm1

*ASA* American society of anesthesiologists, *BMI* Body mass index, *HTN* Hypertension, *DM* Diabetes mellitus, *AF* Atrial fibrillation, *TIA* Transient ischemic attack, *CVI* Cerebral vascular insult

Two board-qualified surgeons performed all operations as partial mesorectal excision (PME), with an anastomosis constructed below the sacral promontory 10–12 cm above the anal verge. Duration of surgery ranged between 152 and 300 min (median 209). The size of the C-REX device was selected intraoperatively, where the 26-mm anastomotic rings were used in fourteen cases, the 29-mm rings in six and the 32 mm-rings in one case.

The six patients operated with the LL-approach had an ACP ranging between 50 and 180 mBar (median 95 mBar). The first patient operated had an ACP of 50 mBar and developed an anastomotic leakage, which was diagnosed and reoperated on postoperative day (POD) 7. The reoperation revealed that the C-REX anastomotic rings were not completely closed manually by the surgeon (Supplementary Fig. 2). After this event and considering the results of the previous CARP study with ACP ranging between 85 and 280 mBar without anastomotic leakage [29], a minimum limit of ACP of 85 mBar was set to proceed with the C-REX technique. Hence, two further patients operated with the LL-approach with an ACP of 50 and 60 mBar, respectively, were converted to conventional stapled anastomoses.

None of the 15 patients operated with the transanal LR-technique developed anastomotic leakage. ACP ranged between 145 and 300 mBar (median 250 mBar). One patient operated laparoscopically was reoperated on POD 7 because of small bowel herniation at one of the port sites, managed through the port hole opening, after which the patient recovered uneventfully. One patient suffered a transient ischemic attack 4 weeks after surgery, and one patient was readmitted 2 weeks after the procedure because of small bowel obstruction that resolved with conservative treatment.

The median time to return of bowel function as indicated by the first defecation was 2 days, with a range of 1–8 days. The C-REX anastomotic rings were spontaneously expelled through the natural route after 7–19 days (median 10 days). Length of stay was 6 days, but the two patients reoperated due to port side hernia and anastomotic leakage were discharge from hospital POD 12 and POD 16 respectively.

**Fig. 4:**
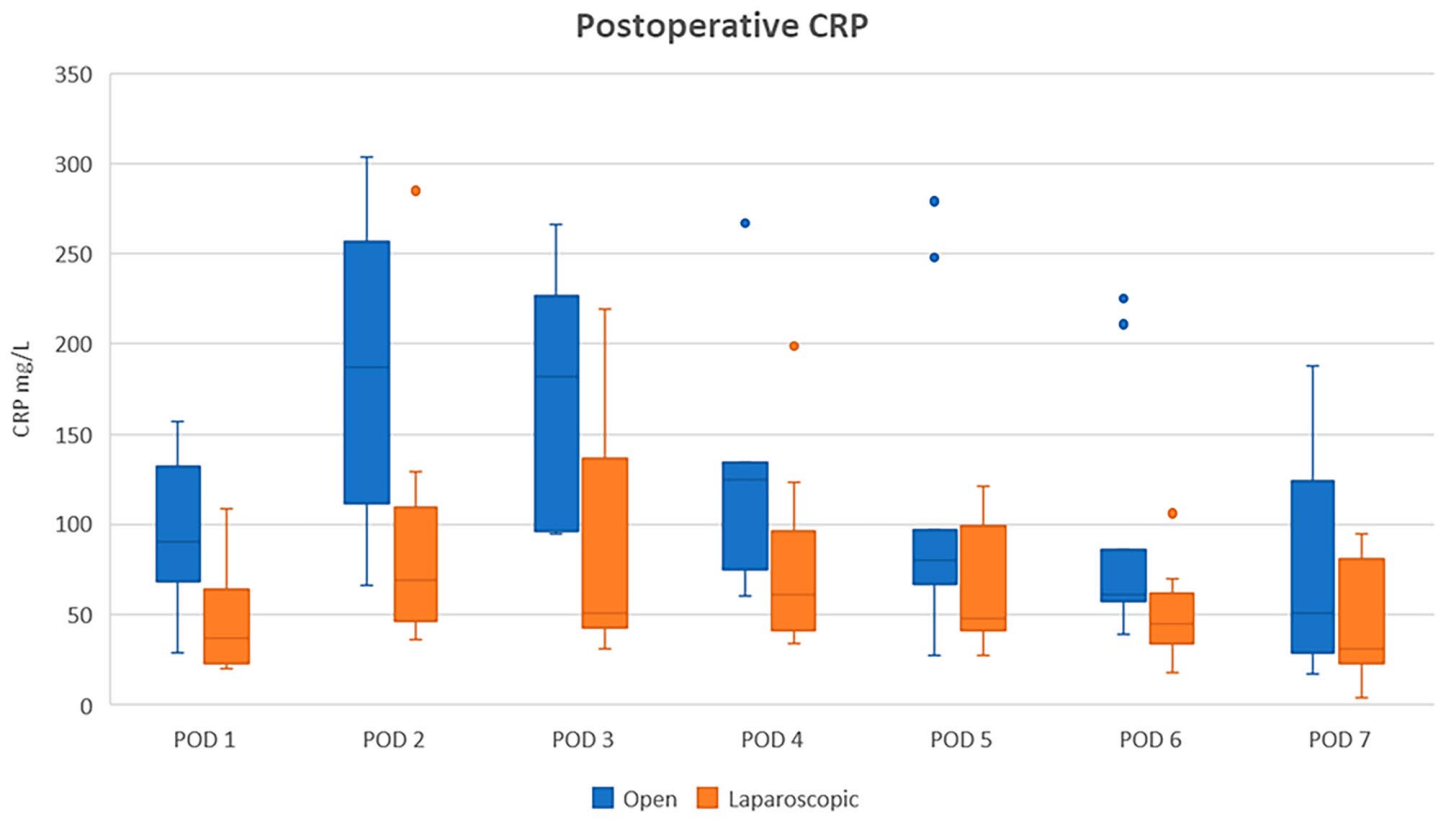
Distribution of the C-reactive protein (CRP) values after open and laparoscopic high anterior resections with C-REX colorectal anastomoses. The distribution is shown with boxes and whiskers plots with range, *Q*_1_, median, *Q*_3_, and IQR. The boxes show 25–75% range. The whiskers show the total range. The horizontal line in the box is the median value. Outliers are shown as dots. There are no extreme values. *POD*, postoperative day

For both open and laparoscopic procedures, CRP and WBC counts peaked on POD 2. Peak CRP values were higher after open surgery with a median CRP of 187 mg/L compared with a median CRP of 69 mg/L following laparoscopic surgery (Fig. 4). Patients with adverse events had biphasic CRP curves compared with uniphasic CRP curves in patient without.

**Fig. 5:**
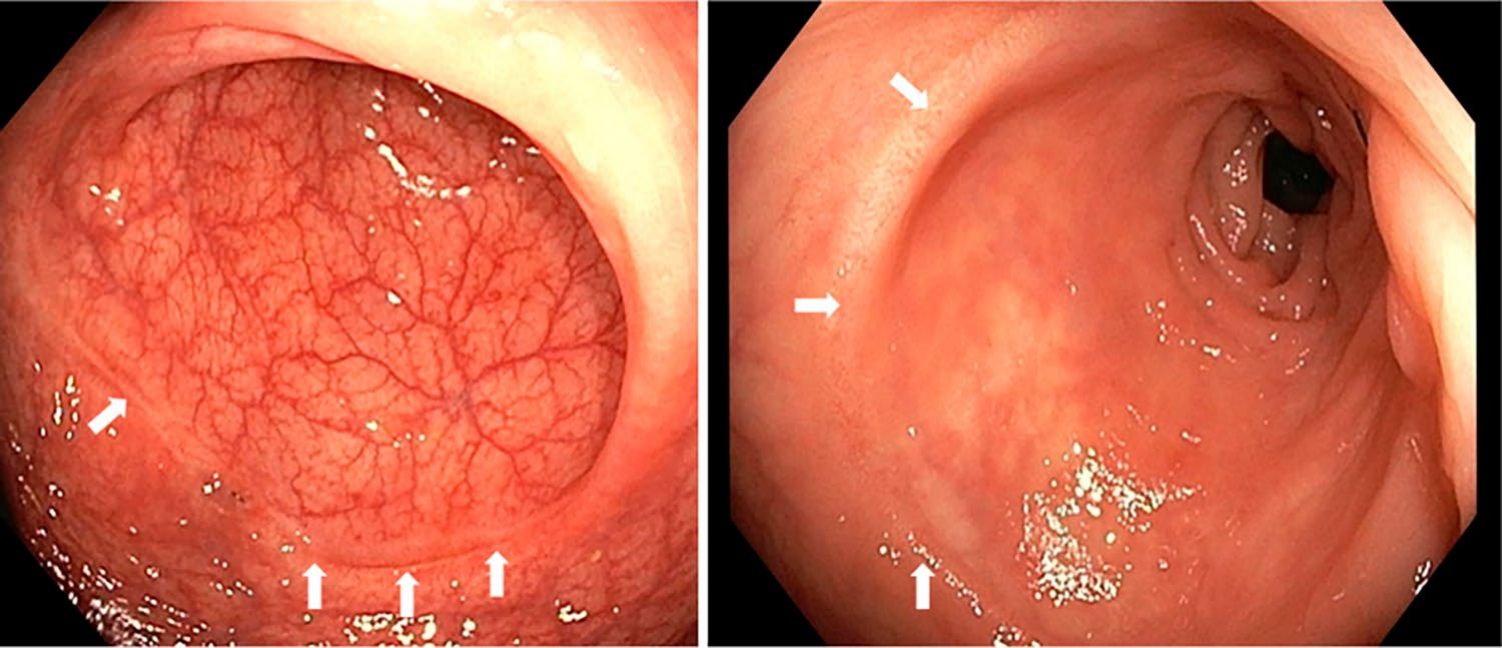
Patients underwent a flexible endoscopy 4–25 weeks after the C-REX surgical procedure (median 12 weeks). This figure shows images of the C-REX anastomosis in two patients, showing a well-healed anastomosis without signs of inflammation or stenosis. The white arrows point at the anastomotic line

Flexible endoscopy was performed in 18 patients with the C-REX anastomosis 4–25 weeks after surgery (median 12 weeks) and showed well-healed anastomoses without signs of pathological inflammation or stenosis in seventeen patients (Fig. 5), whereas one patient had a moderate stricture without any clinical symptoms.

## Discussion

This study evaluated the feasibility and efficacy of the novel C-REX device in colorectal anastomoses following high anterior resections. The C-REX device is based on compression-induced anastomotic healing. This new instrument has been developed based on its predecessor CARP [28, 29]. In contrast to CARP, which was limited to open surgery and colonic anastomosis, C-REX has been developed to enable both laparoscopic surgery and transanal formation of rectal anastomoses. The main finding herein is that transanal formation of anastomoses using C-REX is feasible and effective in patients undergoing high anterior resection.

AL is the most feared complication in colorectal surgery, and the incidence of AL has remained high despite refinement of staple devices and use of diverting stomas [4–15]. The underlying mechanisms of AL are not fully known but are probably multifactorial. One hypothesis has been related to foreign body reaction and anastomotic inflammation [17–20] but also local ischemia and tension created by sutures or staples. To avoid this, the concept of compression-based anastomosis has evolved [21–24]. In fact, compression anastomoses have been shown to be at least comparable to standard suturing and stapling techniques in terms of adverse events in colorectal surgery [21–27]. We have recently reported that CARP is a safe and effective compression device for making colonic anastomosis in patients [29]. C-REX has been developed based on the CARP concept in order to allow laparoscopic and transanal formation of colorectal anastomosis. As with the CARP device, the C-REX technique enables measurement of intraoperative ACP, which provides the surgeon with immediate intraoperative feedback on the compression pressure of the anastomosis.

The ACP was simple to measure via the catheter system of the C-REX device in all the rectal anastomoses in the study. The LL-technique was cumbersome to use for colorectal anastomosis below the sacral promontory, as it requires manual completion by connecting both anastomotic rings together with a click maneuver. To perform this maneuver, both hands of the surgeons must fit into the pelvis. One of the six patients operated with the LL-technique suffered an AL due to inadequate manual compression of the rings. Notably, this patient had an ACP of only 50 mBar. Thus, after this event and considering the results of a previous CARP study, reporting ACP ranging between 85 and 280 mBar without any anastomotic leakage [29], we applied an intraoperative ACP of minimum 85 mBar for all further C-REX anastomosis herein. Two subsequent patients anastomosed with the LL-technique had an ACP of 50 and 60 mBar, respectively, and were consequently converted to stapled colorectal anastomosis. The remaining three patients with an ACP above 85 mBar were anastomosed with the LL-technique and had no anastomotic leakage. This shows the potential of the C-REX system to quantify the intraoperative contact pressure in the anastomosis in order to select optimal surgical strategy.

Due to operator-dependent closure using the LL-technique, we decided to go forward using the more standardized LR-method for transanal formation of anastomoses. The LR-technique uses RectoAid instrument, which resembles traditional circular staplers in design and handling. It was found that ACP was higher than 85 mBar (range 145–300 mBar) in all anastomoses made by use of the LR-method and none was therefore converted to routine stapling. Notably, we observed no case of anastomotic leakage in the group of 15 patients undergoing transanal formation of anastomosis below the sacral promontory. Moreover, no other anastomosis-related complications were observed, such as abscess or fistulas. One patient was however reoperated due to a small bowel herniation at one of the port sites, and another patient suffered from a transient ischemic attack 4 weeks after surgery. Further, one patient was readmitted because of small bowel obstruction that resolved with conservative treatment. Notably, we found endoscopically that all C-REX anastomoses healed well without any signs of pathological inflammation. One patient had a narrow lumen but without clinical symptoms. These results suggest that transanal C-REX is a feasible and effective device for constructing colorectal anastomoses below the sacral promontory in both males and females independent of open or laparoscopic approach. This notion is in line with a previous study showing that CARP is safe and effective in the formation of colonic anastomoses [28, 29]. Future studies will address the potential of transanal C-REX in the formation of low rectal anastomoses.

Taken together, the present study comprises the first cases utilizing the C-REX device in humans in colorectal anastomosis. We found that C-REX was feasible and easy to use although the LL-technique is more cumbersome for anastomoses below the sacral promontory and should be limited to colonic anastomosis. In contrast, the transanal LR-technique is easy for constructing anastomoses below the sacral promontory, and the transanal RectoAid instrument resembles traditional circular staplers in design and handling.

### Supplementary Information

Below is the link to the electronic supplementary material.

Supplementary Fig. 1 This figure shows the steps (A-R) for constructing a transanal anastomosis, where LapAid is used for the proximal bowel end (A-J) and the RectoAid instrument (K-R) is used transanally (LR-technique). RectoAid is similar in appearance to circular stapler devices used in common practice today with an anvil to be placed in the proximal bowel end and connected to the transanally introduced instrument. (PNG 1737 kb)

Supplementary Fig. 2 One patient in the study operated with the LapAid-LapAid technique suffered from an anastomotic leakage. Reoperation revealed that the anastomotic rings were not completely closed together, leaving an inadequate gap between the rings. The image shows the inadequate gap distance on the right side (black arrow) compared to the left side. (PNG 6358 kb)

## Data Availability

Data is available upon request.

## References

[1] Gaidry AD, Tremblay L, Nakayama D, Ignacio RC (2019). The history of surgical staplers: a combination of Hungarian, Russian, and American Innovation. Am Surg.

[2] McGuire J, Wright IC, Leverment JN (1997). Surgical staplers: a review. J R Coll Surg Edinb.

[3] Hardy KJ (1990). Non-suture anastomosis: the historical development. Aust N Z J Surg.

[4] Zbar AP, Nir Y, Weizman A, Rabau M, Senagore A (2012). Compression anastomoses in colorectal surgery: a review. Tech Coloproctol.

[5] Davis B, Rivadeneira DE (2013). Complications of colorectal anastomoses: leaks, strictures and bleeding. Surg Clin North Am.

[6] Kang CY, Halabi WJ, Chaudhry OO, Nguyen V, Pigazzi A, Carmichael JC, Mills S, Stamos MJ (2013). Risk factors for anastomotic leakage after anterior resection for rectal cancer. JAMA Surg.

[7] McDermott FD, Heeney A, Kelly ME, Steele RJ, Carlsson GL, Winter DC (2015). Systematic review of preoperative, intraoperative and postoperative risk factors for colorectal anastomotic leaks. Br J Surg.

[8] Borstlap WAA, Westerduin E, Aukema TS, Bemelman WA, Tanis PJ, Dutch Snapshot Research Group (2017). Anastomotic leakage and chronic presacral sinus formation after low anterior resection: results from a large cross-sectional study. Ann Surg.

[9] Bostrom P, Haapamaki MM, Rutegard J, Matthiessen P, Rutegard M (2018). Population-based cohort study of the impact on postoperative mortality of anastomotic leakage after anterior resection for rectal cancer. BJS Open.

[10] Degiuli M, Elmore U, De Luca R, De Nardi P, Tomatis M, Biondi A, Persiani R, Solaini L, Rizzo G, Soriero D, Cianflocca D, Milone M, Turri G, Rega D, Delrio P, Pedrazzani C, De Palma GD, Borghi F, Scabini S, Coco C, Cavaliere D, Simone M, Rosati R, Reddavid R, Collaborators from the Italian Society of Surgical Oncology Colorectal Cancer Network Collaborative Group (2022). Risk factors for anastomotic leakage after anterior resection for rectal cancer (RALAR study): a nationwide retrospective study of the Italian Society of Surgical Oncology Colorectal Cancer Network Collaborative Group. Colorectal Dis.

[11] Law WL, Choi HK, Lee YM, Ho JWC, Seto CL (2007). Anastomotic leakage is associated with poor long-term outcome in patients after curative colorectal resection for malignancy. J Gastrointest Surg.

[12] Mirnezami A, Mirnezami R, Chandrakumaran K, Sasapu K, Sagar P, Finan P (2011). Increased local recurrence and reduced survival from colorectal cancer following anastomotic leak: systematic review and meta-analysis. Ann Surg.

[13] Wang S, Liu J, Wang S, Zhao H, Ge S, Wang W (2017). Adverse effects of anastomotic leakage on local recurrence and survival after curative anterior resection for rectal cancer: a systematic review and meta-analysis. World J Surg.

[14] Yang J, Chen Q, Jindou L, Cheng Y (2020). The influence of anastomotic leakage for rectal cancer oncologic outcome: a systematic review and meta-analysis. J Surg Oncol.

[15] Koedam TWA, Bootsma BT, Deijen CL, van de Brug T, Kazemier G, Cuesta MA, Fürst A, Lacy AM, Haglind E, Tuynman JB, Daams F, Bonjer HJ (2022). Oncological outcomes after anastomotic leakage after surgery for colon or rectal cancer: increased risk of local recurrence. Ann Surg.

[16] Park JS, Choi GS, Kim SH, Kim HR, Kim NK, Lee KY, Kang SB, Kim JY, Lee KY, Kim BC, Bae BN, Son GM, Lee SI, Kang H (2013). Multicenter analysis of risk factors for anastomotic leakage after laparoscopic rectal cancer excision: the Korean laparoscopic colorectal surgery study group. Ann Surg.

[17] Ballantyne GH (1984). The experimental basis of intestinal suturing. Effect of surgical technique, inflammation, and infection on enteric wound healing. Dis Colon Rectum.

[18] Agren MS, Andersen TL, Mirastschijski U, Syk I, Schiodt CB, Surve V, Lindebjerg J, Delaisse J-M (2006). Action of matrix metalloproteinases at restricted sites in colon anastomosis repair: an immunohistochemical and biochemical study. Surgery.

[19] Lim CB, Goldin RD, Darzi A, Hanna GB (2008). Characterization of materials eliciting foreign body reaction in stapled human gastrointestinal anastomoses. Br J Surg.

[20] Berho M, Wexner SD, Botero-Anug A-M, Pelled D, Fleshman JW (2014). Histopathologic advantages of compression ring anastomosis healing as compared with stapled anastomosis in a porcine model: a blinded comparative study. Dis Colon Rectum.

[21] Kaidar-Person O, Rosenthal RJ, Wexner SD, Szomstein S, Person B (2008). Compression anastomosis: history and clinical consideration. Am J Surg.

[22] Ho YH, Ashour MA (2010). Techniques for colorectal anastomosis. World J Gastroenterol.

[23] Buchberg BS, Masoomi H, Bergman H, Mills SD, Stamos MJ (2011). The use of a compression device as an alternative to hand-sewn and stapled colorectal anastomoses: is three a crowd?. J Gastrointest Surg.

[24] Aggarwal R, Darzi A (2005). Compression anastomoses revisited. J Am Coll Surg.

[25] Hardy TG, Pace WG, Maney JW, Katz AR, Kaganov AL (1985). A biofragmentable ring for sutureless bowel anastomosis. An experimental study. Dis Colon Rectum.

[26] Hardy TG, Aguilar PS, Steward WRC, Katz AR, Maney JW, Costanzo JT, Pace WG (1987). Initial clinical experience with a biofragmentable ring for sutureless bowel anastomosis. Dis Colon Rectum.

[27] Thiede A, Geiger D, Dietz UA, Debus E, Engemann E, Lexer GC, Lünstedt B, Mokros W (1998). Overview on compression anastomoses: biofragmentable anastomosis ring multicenter prospective trial of 1666 anastomoses. World J Surg.

[28] Vilhjalmsson D, Olofsson P, Syk I, Thorlacius H, Grönberg A (2015). Compression anastomotic ring-locking procedure (CARP) is a novel technique for creating a suture-less colonic anastomosis. Eur Surg Res.

[29] Vilhjalmsson D, Appelros A, Toth E, Syk I, Grönberg A, Mynster T, Thorlacius H (2015). Compression anastomotic ring-locking procedure (CARP) is a safe and effective method for intestinal anastomoses following left-sided colonic resection. Int J Colorectal Dis.

