# Supplementary figures and images for: Transanal formation of anastomosis using C-REX device is feasible and effective in high anterior resection
Source: Int J Colorectal Dis. 2023 May 13;38(1):127. doi: 10.1007/s00384-023-04420-x (PMC10182144; doi:10.1007/s00384-023-04420-x)

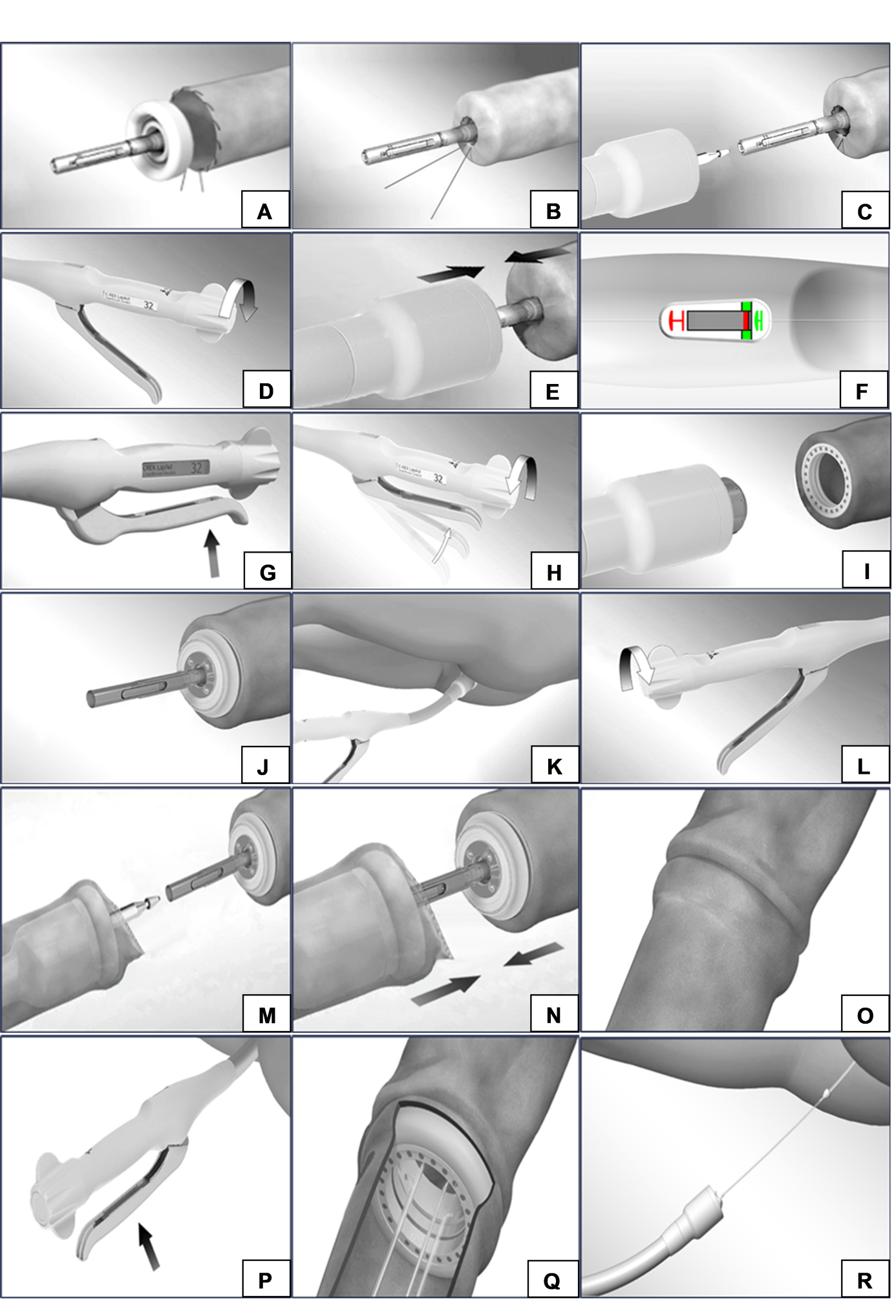

Supplement: Supplementary file 1 — Supplementary Fig. 1 This figure shows the steps (A-R) for constructing a transanal anastomosis, where LapAid is used for the proximal bowel end (A-J) and the RectoAid instrument (K-R) is used transanally (LR-technique). RectoAid is similar in appearance to circular stapler devices used in common practice today with an anvil to be placed in the proximal bowel end and connected to the transanally introduced instrument. (PNG 1737 kb) [file 384_2023_4420_MOESM1_ESM.png]

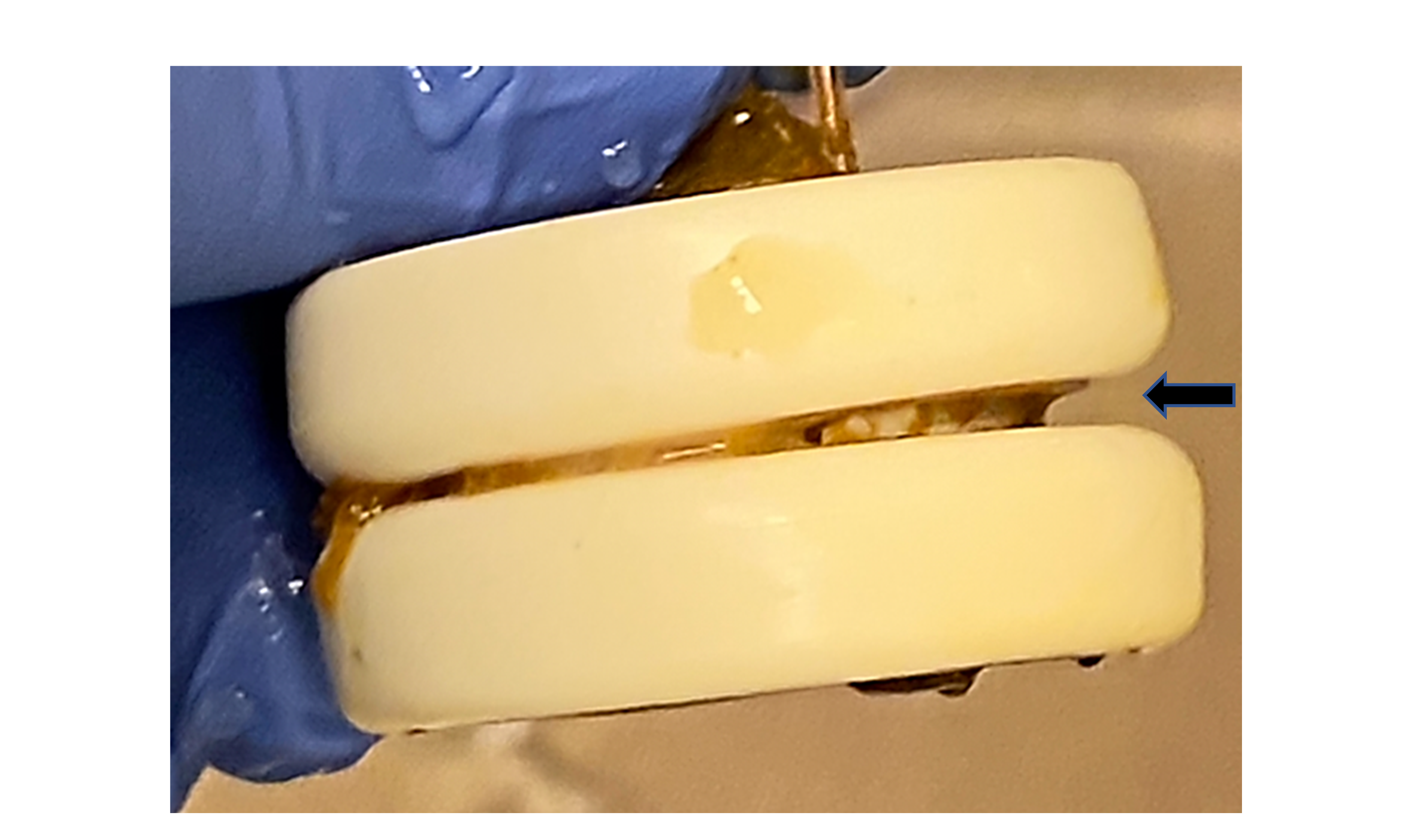

Supplement: Supplementary file 2 — Supplementary Fig. 2 One patient in the study operated with the LapAid-LapAid technique suffered from an anastomotic leakage. Reoperation revealed that the anastomotic rings were not completely closed together, leaving an inadequate gap between the rings. The image shows the inadequate gap distance on the right side (black arrow) compared to the left side. (PNG 6358 kb) [file 384_2023_4420_MOESM2_ESM.png]
